# Dual-strand tumor-suppressor *microRNA-145* (*miR-145-5p* and *miR-145-3p*) coordinately targeted *MTDH* in lung squamous cell carcinoma

**DOI:** 10.18632/oncotarget.12290

**Published:** 2016-09-27

**Authors:** Hiroko Mataki, Naohiko Seki, Keiko Mizuno, Nijiro Nohata, Kazuto Kamikawaji, Tomohiro Kumamoto, Keiichi Koshizuka, Yusuke Goto, Hiromasa Inoue

**Affiliations:** ^1^ Department of Pulmonary Medicine, Graduate School of Medical and Dental Sciences, Kagoshima University, Kagoshima, 890-8520 Japan; ^2^ Department of Functional Genomics, Chiba University Graduate School of Medicine, Chuo-ku, 260-8670 Japan; ^3^ Moores Cancer Center, University of California, San Diego, La Jolla, CA 92093, USA

**Keywords:** microRNA-145-5p, microR-145-3p, tumor-suppressor, MTDH, lung squamous cell carcinoma

## Abstract

Patients with lung adenocarcinoma may benefit from recently developed molecular targeted therapies. However, analogous advanced treatments are not available for patients with lung squamous cell carcinoma (lung SCC). The survival rate of patients with the advanced stage of lung SCC remains poor. Exploration of novel lung SCC oncogenic pathways might lead to new treatment protocols for the disease. Based on this concept, we have identified microRNA- (miRNA) mediated oncogenic pathways in lung SCC. It is well known that *miR-145-5p* (the guide strand) functions as a tumor suppressor in several types of cancer. However, the impact of *miR-145-3p* (the passenger strand) on cancer cells is still ambiguous. Expression levels of *miR-145-5p* and *miR-145-3p* were markedly reduced in cancer tissues, and ectopic expression of these miRNAs inhibited cancer cell aggressiveness, suggesting that both *miR-145-3p* as well as *miR-145-5p* acted as antitumor miRNAs. We identified seven putative target genes (*MTDH*, *EPN3*, *TPD52*, *CYP27B1*, *LMAN1*, *STAT1* and *TXNDC12*) that were coordinately regulated by *miR-145-5p* and *miR-145-3p* in lung SCC. Among the seven genes, we found that metadherin (*MTDH*) was a direct target of these miRNAs. Kaplan–Meier survival curves showed that high expression of *MTDH* predicted reduced survival of lung SCC patients. We investigated pathways downstream from *MTDH* by using genome-wide gene expression analysis. Our data showed that several anti-apoptosis and pro-proliferation genes were involved in pathways downstream from *MTDH* in lung SCC. Taken together, both strands of *miR-145*, *miR-145-5p* and *miR-145-3p* are functional and play pivotal roles as antitumor miRNAs in lung SCC.

## INTRODUCTION

Lung cancer is the most frequent cause of cancer-related death worldwide [1]. Non-small cell lung cancer (NSCLC) is the most common type of lung cancer and is divided into three subtypes according to pathogenesis: adenocarcinoma, squamous cell carcinoma and large cell carcinoma [2]. Recent developments in molecular targeted therapies have improved the overall survival of patients with lung adenocarcinoma [3]. In contrast, there is a lack of effective therapeutic options to treat patients with lung squamous cell carcinoma (lung SCC) [3]. In that regard, cancer cell metastasis is known to be an important prognostic indicator of lung cancer. Thus, we hypothesized that current genomic approaches might be used to elucidate the molecular mechanisms underlying lung SCC metastasis and suggest improved treatments for this disease.

MicroRNA (miRNA) is a class of small non-coding RNAs. They are involved in the repression or degradation of target RNA transcripts in a sequence-dependent manner [4–6]. Aberrantly expressed miRNAs can dysregulate cellular RNA networks critical for normal cell function. The resultant failure of RNA networks promotes malignant transformation of cancer cells [7–9]. Identification of aberrantly expressed miRNAs is the first step to defining the oncogenic RNA networks in cancer cells. With that in mind, we used clinical specimens to construct miRNA expression signatures of various types of human cancer [10–14]. Using these signatures, we identified antitumor miRNAs and miRNA-regulated oncogenic genes [10–14]. Our recent studies of lung SCC revealed that *miR-1/miR-133a* clustered miRNAs, *miR-206* and the *miR-29-family* inhibited cancer cell migration and invasion through targeting of several oncogenic genes, such as coronin-1C (*CORO1C*), *c-MET* and lysyl oxidase like 2 (*LOXL2*) [15–17].

Using miRNA expression signatures obtained by deep sequencing, we found that expression levels of *miR-145-5p* (the guide strand of *pre-miR-145*) and *miR-145-3p* (the passenger strand of *pre-miR-145*) were significantly reduced in cancer tissues, suggesting that these miRNAs functioned as tumor-suppressors [18]. It is believed that the guide strand RNA from duplex miRNA is retained to direct recruitment of the RNA-induced silencing complex (RISC) to target messenger RNAs. In contrast, the passenger strand RNA is degraded in the course of miRNA biogenesis [19]. Our recent study overturned this convention. We found that both the *miR-144-5p* strand and the *miR-144-3p* strand derived from *pre-miR-144* acted as tumor suppressors in bladder cancer (BC) cells [20]. Moreover, we showed that *miR-145-3p* acted as a tumor-suppressor in BC cells, indicating that the passenger strand of miRNA has pivotal roles in human cancer pathogenesis [18].

Downregulation of *miR-145-5p* was reported in several cancers, establishing its function as a tumor-suppressor [21–26]. However, the role of *miR-145-3p* on lung cells is still ambiguous. The aims of the present study were to investigate the anti-tumor effects of *miR-145-3p* as well as *miR-145-5p* in lung cells. We also sought to identify oncogenic RNA networks in lung SCC and the genes regulated by these miRNAs. Our present data showed that *miR-145-3p* functions as a tumor-suppressor as well as *miR-145-5p* in lung SCC cells. Moreover, gene expression data and *in silico* database analysis showed that the metadherin gene (*MTDH*), also known as astrocyte elevated gene-1 (*AEG-1*) was a direct target of both *miR-145-5p* and *miR-145-3p* regulation. Kaplan–Meier survival curves showed that high expression of *MTDH* predicted poorer survival of lung SCC patients. The discovery of new tumor-suppressor functions of both miRNAs strands of *miR-145* (*miR-145-5p* and *miR-145-3p*) provides new insight into the molecular mechanisms of lung SCC pathogenesis.

## RESULTS

### Expression levels of *miR-145-5p* and *miR-145-3p* in lung SCC clinical specimens and cell lines

**Figure 1:**
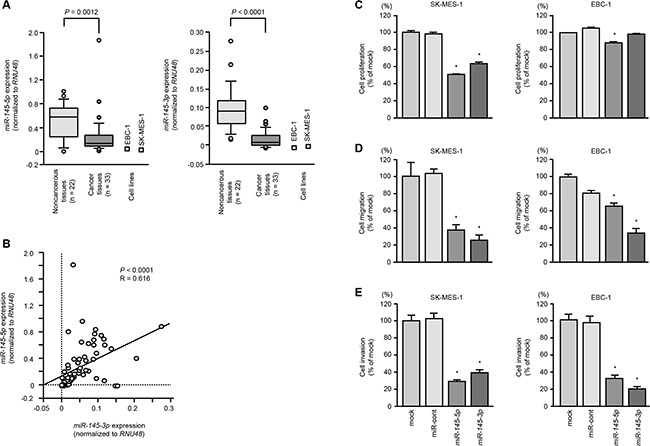
The expression levels of *miR-145-5p* and *miR-145-3p* in lung SCC cells and their ectopic effects in cancer cells **A.** Expression levels of *miR-145-5p* and *miR-145-3p* in lung SCC clinical specimens and cell lines (SK-MES-1 and EBC-1) were determined by qRT-PCR. Data were normalized to *RNU48* expression. **B.** Correlation of the expression levels of *miR-145-5p* and *miR-145-3p*. **C.** Cell growth was determined by XTT assays 72 h after transfection with 10 nM *miR-145-5p* or *miR-145-3p*. *P < 0.05. **D.** Cell migration activity was determined by wound healing assays. *P < 0.001. **E.** Cell invasion activity was determined using Matrigel invasion assays. *P < 0.001.

We evaluated the expression levels of dual strand miRNAs of *pre-miR-145* (*miR-145-5p* and *miR-145-3p)* in lung SCC tissues. The expression levels of *miR-145-5p* and *miR-145-3p* were significantly reduced in lung SCC tissues compared to noncancerous tissues (*P* = 0.0012 and *P* < 0.0001, respectively, Figure 1A). Spearman's rank test showed positive correlations between the expression of *miR-145-5p* and *miR-145-3p* (R = 0.616 and *P* < 0.0001; Figure 1B).

The patients’ backgrounds and clinicopathological characteristics are summarized in Table 1A. Normal lung tissues are summarized in Table 1B. There were no significant relationships between any of the clinicopathological parameters (i.e., tumor grade, stage, metastasis, or survival rate) and the expression levels of *miR-145-5p* and *miR-145-3p* (data not shown).

**Table 1A:** Characteristic of patients

Lung cancer		SCC	(%)
Total number		33	
Median age (range)		70 (50 - 88)	
Gender			
	Male	31	(93.9)
	Female	2	(6.1)
Pathological tumor stage			
	IA	5	(15.2)
	IB	8	(24.2)
	IIA	4	(12.1)
	IIB	6	(18.2)
	IIIA	8	(24.2)
	IIIB	1	(3.0)
	unknown	1	(3.0)
Differentiation			
	well	8	(24.2)
	moderately	20	(60.6)
	poorly	3	(9.1)
	unknown	2	(6.1)
Pleural invasion			
	(+)	15	(45.5)
	(−)	18	(54.5)
Venous invasion			
	(+)	16	(48.5)
	(−)	17	(51.5)
Lymphatic invasion			
	(+)	16	(48.5)
	(−)	17	(51.5)

**Table 1B:** Characteristic of Patients

Normal lung		n
Total number		22
Median age (range)		71 (50 - 88)
Gender		
	Male	22
	Female	0

### Effects of ectopic expression of *miR-145-5p* or *miR-145-3p* on cell proliferation, migration and invasion in SK-MES-1 and EBC-1 cells

To investigate the functional roles of *miR-145-5p* and *miR-145-3p*, we performed gain-of-function studies using mature miRNA transfections of SK-MES-1 and EBC-1 cells. The expression levels of *miR-145-5p* and *miR-145-3p* were significantly lower in two cell lines (Figure 1A).

XTT assays revealed significant inhibition of cell proliferation in SK-MES-1 cells transfected with *miR-145-5p* or *miR-145-3p* in comparison with mock or control transfectants (*P* < 0.05, Figure 1C). EBC-1 cells transfected with *miR-145-3p,* there was no significant inhibition of cell proliferation in comparison with control transfectants (Figure 1C).

Wound healing assays showed significant inhibition of cell migration activity after transfection with *miR-145-5p* or *miR-145-3p* (*P* < 0.001, Figure 1D).

Similarly, Matrigel invasion assays revealed that transfection with *miR-145-5p* of *miR-145-3p* reduced cell invasion activities (*P* < 0.001, Figure 1E).

To investigate the synergistic effects of *miR-145-5p* and *miR-145-3p,* we performed functional assays (cell proliferation, migration and invasion assays) with co-transfection of mature *miR-145-5p* and *miR-145-3p* in EBC-1 cells. In this assays, we did not detected the synergistic effects by using these miRNAs transfection (Supplementary Figure S1)

### Identification of target genes coordinately regulated by *miR-145-5p* and *miR-145-3p* in lung SCC cells

To identify target genes coordinately regulated by *miR-145-5p* and *miR-145-3p*, we performed a combination of *in silico* analyses, oligomicroarray expression analyses and Gene Omnibus database (GEO) analyses.

The TargetScan database showed that 4,405 and 3,164 genes have putative target sites for *miR-145-5p* and *miR-145-3p* in their 3′UTRs, respectively. Next, we performed genome-wide gene expression analysis using EBC-1 cells (GEO accession number GSE77790). Genes downregulated (log_2_ ratio < -1.0) by transfection with *miR-145-5p* or *miR-145-3p* were selected as putative target genes. A total of 314 and 155 genes were downregulated in *miR-145-5p* and *miR-145-3p* transfectants, respectively. We found that there were 13 common genes targeted by both miRNAs. Finally, to evaluate upregulated genes in clinical NSCLC specimens, we examined gene expression profiles in the GEO database (accession numbers GSE19188).

**Figure 2:**
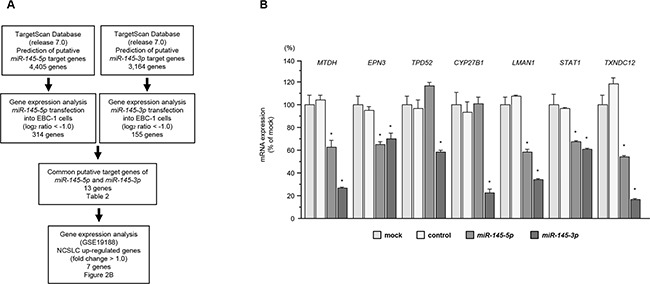
Identification of genes coordinately regulated by *miR-145-5p* and *miR-145-3p* **A.** Flow chart illustrates the strategy for analysis of *miR-145-5p* and *miR-145-3p* target genes in lung SCC cells. **B.** Identification of target genes of *miR-145-5p* or *miR-145-3p*. Expression levels of 7 mRNAs (*MTDH*, *EPN3*, *TPD52*, *CYP27B1*, *LMAN1*, *STAT1* and *TXNDC12*) were evaluated by qRT-PCR in EBC-1 cells 72 h after transfection with *miR-145-5p* or *miR-145-3p*. *GUSB* was used as an internal control. *P < 0.0001.

A total of 7 putative candidate genes for both *miR-145-5p* and *miR-145-3p* regulation were identified (Table 2). A flow chart describing the strategy for analysis of *miR-145-5p* and *miR-145-3p* target genes is shown in Figure 2A. We examined real-time RT-qPCR analyses of EBC-1 cells to investigate whether restoration of *miR-145-5p* or *miR-145-3p* expression altered the expression of 7 genes mRNA. The mRNA expression levels of 7 candidate genes were shown in Figure 2B. Among these genes, *MTDH*, *EPN3*, *LMAN1*, *STAT1*, and *TXNDC12* were significantly repressed in *miR-145-5p* transfectants as compared with mock- or miR-control-transfected cells. All candidate target genes were significantly repressed in *miR-145-3p* transfectants as compared with mock- or miR-control-transfected cells.

**Table 2:** Downregulated genes in *miR-145-5p/3p* transfectant

Entrez Gene ID	common target	Gene name	*miR-145-5p*		*miR-145-3p*	*miR-145-5p* transfection (log_2_)	*miR-145-3p* transfection (log_2_)	GSE19188 (fold-change)
			conserved	poorly conserved	poorly conserved			
92140	*MTDH*	*metadherin*	1	1	1	−1.03	−1.34	1.29
55040	*EPN3*	*epsin 3*	0	1	1	−1.00	−1.28	6.01
7163	*TPD52*	*tumor protein D52*	0	2	1	−1.18	−1.26	2.66
1594	*CYP27B1*	*cytochrome P450, family 27, subfamily B, polypeptide 1*	0	1	2	−1.74	−1.40	2.12
79748	*LMAN1*	*lectin, mannose-binding, 1*	0	1	1	−1.08	−1.14	1.78
6772	*STAT1*	*signal transducer and activator of transcription 1, 91kDa*	0	1	1	−1.30	−1.01	1.61
51060	*TXNDC12*	*thioredoxin domain containing 12 (endoplasmic reticulum)*	0	1	1	−2.13	−2.72	1.20

We did not detected the synergistic effects (co-transfection of *miR-145-5p* and *miR-145-3p*) for expression status of *MTDH* in SK-MES-1 and EBC-1 cells (Supplementary Figure S2).

### *MTDH* was direct target by *miR-145-5p* and *miR-145-3p* in lung SCC cells

**Figure 3:**
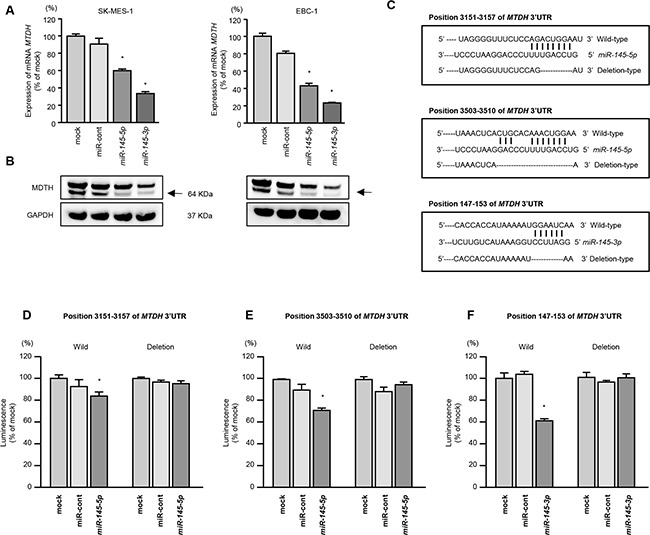
Direct regulation of *MTDH* by *miR-145-5p* or *miR-145-3p* in lung SCC cells **A.** *MTDH* mRNA expression was evaluated by qRT-PCR in SK-MES-1 and EBC-1 cells 72 h after transfection with *miR-145-5p* or *miR-145-3p*. *GUSB* was used as an internal control. *P < 0.0001. **B.** MTDH protein expression was evaluated by Western blot analyses in SK-MES-1 and EBC-1 72 h after transfection with *miR-145-5p* or *miR-145-3p*. GAPDH was used as a loading control. **C.** *miR-145-5p* or *miR-145-3p* binding sites in the 3′-UTR of *MTDH* mRNA. (D-F) Dual luciferase reporter assays using vectors encoding putative *miR-145-5p* (positions 3,151-3,157 or 3,503-3,510) or *miR-145-3p* (147-153) target sites of the *MTDH* 3′-UTR for both wild-type and deleted regions. Normalized data were calculated as ratios of *Renilla*/firefly luciferase activities. *P = 0.0054, *P* < 0.001, and *P* < 0.001 for **D-F.**, respectively.

We performed real-time RT-qPCR and Western blotting analyses of SK-MES-1 and EBC-1 cells to investigate whether restoration of *miR-145-5p* or *miR-145-3p* expression altered the expression of *MTDH* mRNA and MTDH protein. The mRNA and protein expression levels of *MTDH*/MTDH were significantly repressed in *miR-miR-145-5p* or *miR-145-3p* transfectants as compared with mock- or miR-control-transfected cells (Figure 3A and 3B).

We next performed luciferase reporter assays using EBC-1 cells to determine whether *MTDH* mRNA had target sites for *miR-145-5p* and *miR-145-3p*. The TargetScan database identified two putative target sites in the 3′-UTR of *MTDH* for *miR-145-5p* (position 3151-3157 and position 3503-3510) and one site for *miR-145-3p* (position 147-153) (Figure 3C). We used vectors encoding a partial wild-type sequence of the 3′-UTR of MTDH mRNA, including the predicted *miR-145-5p* and *miR-145-3p* target site, or a vector lacking the *miR-145-5p* and *miR-145-3p* target sites. We found that the luminescence intensity was significantly reduced by co-transfection with *miR-145-5p* or *miR-145-3p* and the vector carrying the wild-type 3′-UTR of *MTDH*. On the other hand, the luminescence intensity was not decreased when the seed sequences of the target sites were deleted from the vectors (*P* = 0.0054, *P* < 0.001, and *P* < 0.001, respectively; Figures 3D-3F).

### Effects of silencing *MTDH* on cell aggressiveness in lung SCC cells

**Figure 4:**
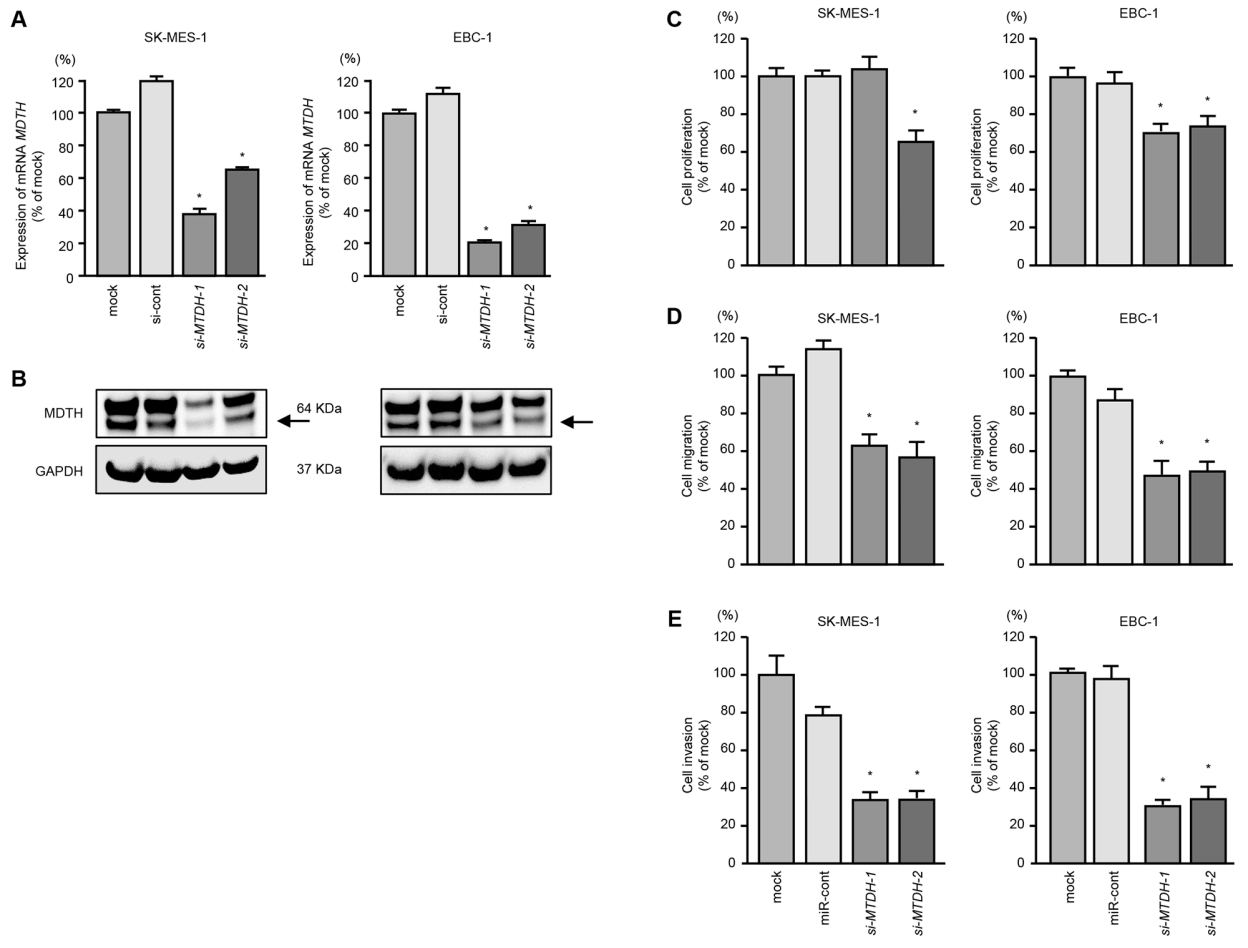
Effects of *MTDH* silencing in lung SCC cell lines **A.** *MTDH* mRNA expression was evaluated by qRT-PCR in SK-MES-1 and EBC-1 72 h after transfection with *si-MTDH*-1 or *si-MTDH*-2. *GUSB* was used as an internal control. **B.** MTDH protein expression was evaluated by Western blot analysis in SK-MES-1 and EBC-1 72 h after transfection with *miR-145-5p* or *miR-145-3p*. GAPDH was used as a loading control. **C.** Cell proliferation was determined with the XTT assays 72 h after transfection with 10 nM si-*MTDH*-1 or si-*MTDH*-2. *P < 0.05. **D.** Cell migration activity was determined by wound healing assays. *P < 0.001. **E.** Cell invasion activity was determined using Matrigel invasion assays. *P < 0.001.

We investigated the oncogenic function of *MTDH* in lung SCC cells by using si-*MTDH* transfectants. We evaluated the knockdown efficiency of si-*MTDH* transfection in lung SCC cells. Present data indicated that si-*MTDH* effectively downregulated *MTDH/*MTDH expression in lung SCC cells (Figure 4A and 4B).

Cell proliferation was inhibited by si-*MTDH* transfectants in comparison with mock or si-control transfectants in EBC-1 cells (*P* < 0.05, Figure 4C). However in SK-MES-1 cells transfected with si-*MTDH*-1, proliferation was not inhibited in comparison with mock or si-control transfectants (Figure 4C).

Wound healing and Matrigel invasion assays showed significant inhibition of cell migration and invasion by si-*MTDH* transfectants (*P* < 0.001, Figures 4D and 4E).

### Expression of *MTDH* in clinical lung SCC specimens

**Figure 5:**
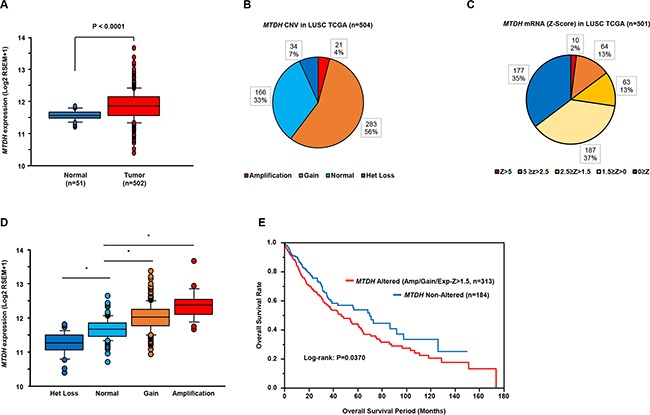
Clinical significance of *MTDH* expression in lung SCC based on TCGA database **A.** Comparison of *MTDH* mRNA expression levels between normal and tumor samples. **B, C.** The distribution of *MTDH* genomic copy number variation (n = 504) and mRNA Z-score (n = 501) in LUSCC TCGA. **D.** Box-and-whisker plots of *MTDH* mRNA expression with respect to genomic copy number *P < 0.0001. **E.** Clinical outcome for patients with altered *MTDH* (CNV, amplification or gain, or mRNA Z-score > 1.5) or non-altered *MTDH* (CNV, diploid or het loss, and mRNA Z-score ≤ 1.5) are displayed as Kaplan–Meier plots with log-rank tests.

The mRNA expression levels of *MTDH* were significantly upregulated in lung SCC clinical samples (Figure 5A). As for *MTDH* copy number variation (CNV), our study showed that 60% of lung SCC patients had a genetic alteration (Figure 5B and 5C). The mRNA expression levels of *MTDH* significantly increased in accordance with the increase of *MTDH* gene copy number (Figure 5D). We assessed the Kaplan–Meier univariate survival of patients groups, comparing those with an *MTDH* alteration (CNV amplification or gain, or mRNA Z-score > 1.5) and those without an *MTDH* alteration (diploid or heterozygous loss, and mRNA Z-score equal or less than 1.5). The *MTDH* altered group had a significantly poorer overall survival [*P* = 0.0370] (Figure 5E).

### Identification of *MTDH*-mediated downstream pathways in lung SCC cells

To identify the downstream genes regulated by *MTDH*, genome-wide gene expression analyses and *in silico* analyses were performed in lung SCC cells transfected with *si-MTDH*. Our present expression data using EBC-1 cells was deposited in GEO database (GEO accession number: GSE82108).

A total of 1,841 genes were reduced in si-*MTDH* transfected cells. Downregulated genes (top 40 genes) were listed in Supplementary Tables S1A and S1B. Furthermore, to investigate the functional roles of *MTDH*-mediated genes, we categorized these downregulated genes by KEGG pathways. Genes involved in these pathways are listed in Supplementary Table S2. Among these pathways, we focused on “Focal adhesion”, “Pathways in cancer” and “Endocytosis” pathways because *MTDH* contributed to cancer cell migration and invasion (Table 3).

**Table 3A:** Downregulated genes by si-*MTDH* in “Focal adhesion pathway”

Entrez gene ID	Gene symbol	Description	si-*MTDH* transfectant (fold-change)		GSE19188 (fold-change)
			siRNA1	siRNA2	
1290	*COL5A2*	collagen, type V, alpha 2	0.02	−0.07	3.55
3688	*ITGB1*	integrin, beta 1	−2.28	−0.05	2.64
2317	*FLNB*	filamin B, beta	−2.20	−2.01	2.43
7422	*VEGFA*	vascular endothelial growth factor A	−2.06	0.36	1.99
3915	*LAMC1*	laminin, gamma 1 (formerly LAMB2)	−3.22	0.18	1.61
1956	*EGFR*	epidermal growth factor receptor	−2.30	−0.88	1.50
3371	*TNC*	tenascin C	−4.17	−0.37	1.46
5501	*PPP1CC*	protein phosphatase 1, catalytic subunit, gamma isozyme	−0.60	−2.04	1.42
5500	*PPP1CB*	protein phosphatase 1, catalytic subunit, beta isozyme	−4.54	−2.17	1.37
10000	*AKT3*	v-akt murine thymoma viral oncogene homolog 3	−2.73	−2.19	1.37
394	*ARHGAP5*	Rho GTPase activating protein 5	−2.63	0.00	1.36
208	*AKT2*	v-akt murine thymoma viral oncogene homolog 2	0.22	−2.70	1.34
5578	*PRKCA*	protein kinase C, alpha	−0.26	−3.17	1.30
3914	*LAMB3*	laminin, beta 3	−2.31	−0.20	1.04

**Table 3B:** Downregulated genes by si-*MTDH* in “Pathways in cancer”

Entrez gene ID	Gene symbol	Description	si-*MTDH* transfectant (fold-change)		GSE19188 (fold-change)
			siRNA1	siRNA2	
6513	*SLC2A1*	solute carrier family 2 (facilitated glucose transporter), member 1	−2.02	−0.30	6.34
112399	*EGLN3*	egl-9 family hypoxia-inducible factor 3	−2.67	−0.48	5.97
5888	*RAD51*	RAD51 recombinase	−1.08	1.16	4.24
4318	*MMP9*	matrix metallopeptidase 9 (gelatinase B, 92kDa gelatinase, 92kDa type IV collagenase)	−0.02	−2.41	4.12
1163	*CKS1B*	CDC28 protein kinase regulatory subunit 1B	−2.27	0.87	3.42
5337	*PLD1*	phospholipase D1, phosphatidylcholine-specific	−0.82	−2.39	2.83
3688	*ITGB1*	integrin, beta 1	−2.28	−0.05	2.64
5578	*PRKCA*	protein kinase C, alpha	−0.26	−3.17	2.08
7184	*HSP90B1*	heat shock protein 90kDa beta (Grp94), member 1	−2.88	0.03	2.00
7422	*VEGFA*	vascular endothelial growth factor A	−2.06	0.05	1.99
8030	*CCDC6*	coiled-coil domain containing 6	−7.46	0.65	1.62
3915	*LAMC1*	laminin, gamma 1 (formerly LAMB2)	−3.22	0.18	1.61
54205	*CYCS*	cytochrome c, somatic	−2.20	1.34	1.59
1956	*EGFR*	epidermal growth factor receptor	−2.30	−0.88	1.50
613	*BCR*	breakpoint cluster region	−2.43	−0.76	1.28
208	*AKT2*	v-akt murine thymoma viral oncogene homolog 2	0.22	−2.70	1.34
10000	*AKT3*	v-akt murine thymoma viral oncogene homolog 3	−2.73	−2.19	1.37
5728	PTEN	*phosphatase and tensin homolog*	0.13	−3.01	1.26
861	*RUNX1*	runt-related transcription factor 1	−3.21	−3.90	1.26
3914	*LAMB3*	laminin, beta 3	−2.31	−0.20	1.04

**Table 3C:** Downregulated genes by si-*MTDH* in “Endcytosis pathway”

Entrez gene ID	Gene symbol	Description	si-*MTDH* transfectant (fold-change)		GSE19188 (fold-change)
			siRNA1	siRNA2	
55040	*EPN3*	epsin 3	0.93	−2.86	6.01
57154	*SMURF1*	SMAD specific E3 ubiquitin protein ligase 1	−2.20	−0.07	2.71
83737	*ITCH*	itchy E3 ubiquitin protein ligase	−2.20	−0.67	2.42
163	*AP2B1*	adaptor-related protein complex 2, beta 1 subunit	−2.28	−0.99	1.76
9815	*GIT2*	G protein-coupled receptor kinase interacting ArfGAP 2	0.06	−2.30	1.53
51100	*SH3GLB1*	SH3-domain GRB2-like endophilin B1	−3.11	−2.15	1.11
9922	*IQSEC1*	IQ motif and Sec7 domain 1	−0.14	−2.90	1.35
2869	*GRK5*	G protein-coupled receptor kinase 5	−0.19	−2.14	1.35

## DISCUSSION

In miRNA biogenesis, processing of the pre-miRNA through Dicer1 generates a miRNA duplex (a passenger strand and a guide strand). The guide strand RNA from the duplex is retained and recruited to the RNA induced silencing complex (RISC) to target messenger RNAs, whereas the passenger strand has no regulatory activity and is degraded [27, 28]. Our recent studies showed that passenger strands of some miRNAs (*miR-144-3p* and *miR-145-3p*) have antitumor functions and directly target several oncogenic genes in BC cells [18, 20]. In this study, we demonstrated that *miR-145-3p* (passenger strand) had antitumor functions as did *miR-145-5p* (guide strand) in lung SCC cells. A large number of studies showed the antitumor function of *miR-145-5p* in several cancers, including lung cancer [21–26]. Based on previous studies and our data, both strands of *pre-miR-145* (*miR-145-5p* and *miR-145-3p*) act as antitumor miRNAs in cancer cells.

We hypothesized that *miR-145-5p* and *miR-145-3p* coordinately regulated target genes that significantly contributed to lung SCC pathogenesis. In this study, we applied *in silico* and gene expression analyses described in our previous studies [15–17, 18, 20]. Using that strategy, we previously found that ubiquitin-like with PHD and ring finger domains 1 (*UHRF1*) was directly regulated by both *miR-145-5p* and *miR-145-3p* in BC [18]. Moreover, we showed that *UHRF1* was overexpressed in BC clinical specimens and that the high *UHRF1* expression group had a significantly poorer cause-specific survival rate in comparison with the low expression group [18].

Here, we identified 7 putative candidate genes (*MTDH*, *EPN3*, *TPD52*, *CYP27B1*, *LMAN1*, *STAT1* and *TXNDC12*) that were regulated by both *miR-145-5p* and *miR-145-3p* in lung SCC cells. Several studies showed that *TPD52* was overexpressed in several cancers, and function as an oncogenes [29, 30]. Recent study indicated that *miR-145-5p* and its target gene *TPD52* contributed to malignancy progression and in metastasis of brain tumor [31]. In colon cancer cells, overexpression of *miR-145-5p* reduced *STAT1* expression [32]. These facts suggest that list of candidate genes might be regulated by both *miR-145-5p* and *miR-145-3p* in lung cancer cells. These genes mediated cancer pathways that are important for understanding lung SCC pathogenesis. We focused on *MTDH* in the present study and investigated its functional significance in lung SCC. *MTDH* is also termed astrocyte elevated gene-1 (*AEG-1*) and lysine-rich CEACA-M1 co-isolated (*LYRIC*). It was initially cloned from human fetal astrocytes following HIV-1 infection or tumor necrosis factor-alpha treatment [33]. The function of *MTDH* is a downstream mediator of several signal pathways, such as PI3K/AKT, NFκB, MAPK and Wnt/β-catenin [34, 35]. These activated pathways are deeply involved in cancer cells proliferation, invasion, angiogenesis and metastasis [34, 35]. Our function assays showed that knockdown of *MTDH* inhibited cancer cell aggressiveness and affected downstream signal pathways under the following headings: “focal adhesion”, “pathways in cancer”, “endocytosis” and “cell cycle”. Our findings in this study support the oncogenic function of *MTDH* in lung SCC cells.

The primary finding of the present study is the overexpression of *MTDH,* a gene that is involved in the pathogenesis of lung SCC. Our large cohort study indicated that expression of *MTDH* was upregulated in cancer tissues. Furthermore, Kaplan–Meier survival curves showed that high expression of *MTDH* predicted poorer survival of lung SCC patients. Our functional study showed that silencing of *MTDH* inhibited lung-SCC cell migration and invasion. Metastasis is responsible for most of the mortality in lung cancer. Other studies demonstrated that high level *MTDH* expression predicted poor survival of patients with esophageal squamous cell carcinoma and breast cancer [35–38].

Recent studies showed that several miRNAs control the expression of *MTDH* in multiple types of cancer, such as *miR-375*, *miR-136*, *miR-26a* and *miR-145-5p* [39–44]. Downregulation of these miRNAs enhanced the expression of *MTDH* in cancer cells. Several studies indicated that activation of *H-Ras*/*PI3K* signaling induce the expression of MYC, which binds to the *MTDH* promoter region and enhances its expression [34, 45]. Interestingly, p53 appears to transcriptionally regulate *miR-145-5p* by its interaction with a potential p53 response element in the *pre-miR-145* promoter region and *miR-145-5p* directly targets oncogenic MYC [46, 47]. In the human genome, *MTDH* is located in 8q22. This area is associated with the center of activity for genomic amplification in multiple cancers [48]. Recent study showed that *MTDH* and *MYC* cooperate to promote hepatic cancer [49]. Thus, it appears that overexpression of *MTDH* in cancer cells enhances its aggressiveness.

In conclusion, downregulation of dual strand *pre-miR-145* (*miR-145-5p* and *miR-145-3p*) was detected in lung SCC clinical specimens. Both *miR-145-3p* and *miR-145-5p* act as antitumor miRNAs in lung SCC cells. Oncogenic *MTDH* was directly regulated by these miRNAs. Expression of *MTDH* is involved in lung SCC pathogenesis and its high expression predicts poorer survival of lung SCC patients. Elucidation of *miR-145-5p/miR-145-3p/MTDH*-mediated molecular networks may lead to a better understanding of lung SCC pathogenesis and the development of new treatment protocols.

## MATERIALS AND METHODS

### Clinical specimens, cell culture and RNA extraction

From 2010 to 2013, Kagoshima University Hospital collected 33 lung SCCs and 22 noncancerous lung specimens from patients who underwent pneumonectomy. Our study was approved by the Institutional Review Board for Clinical Research of Kagoshima University School of Medicine. Each patient gave us prior written informed consent and approval.

We used the International Association for the Study of Lung Cancer TNM classification system to stage the samples. All samples were histologically graded [50].

Archival formalin-fixed paraffin embedded (FFPE) samples were used for qRT-PCR analysis. Eight FFPE tissue sections (10-μm) were used for extraction of total RNA, using Recover All™ Total Nucleic Acid Isolation kits Ambion (Texas, USA) according to the manufacturer's protocols.

Two human lung SCC cell lines (SK-MES-1 and EBC-1) were obtained from Japanese Cancer Research Resources Bank (JCRB) and the American Type Culture Collection (Manassas, VA, USA), respectively.

### Quantitative real-time PCR (qRT-PCR)

PCR quantification was carried out essentially as previously described [12–17]. TaqMan probes and primers for *MTDH* (assay ID: Hs00757841_m1, Applied Biosystems, Foster City, CA, USA), *EPN3* (HS00978957_m1), *TPD52* (Hs00893105_m1), *CYP27B1* (Hs01096154_m1), *LMAN1* (Hs01557542), *STAT1* (Hs01013996_m1) and *TXNDC12* (Hs00210841_m1) were assay-on-demand gene expression products. To quantify the expression level of miRNAs, we utilized stem-loop RT-PCR for *miR-145-5p* (assay ID: 002278, Applied Biosystems) and *miR-145-3p* (assay ID: 002149, Applied Biosystems) following the manufacturer's protocol. mRNA and miRNA data were normalized to human *GUSB* (assay ID: Hs99999908_m1; Applied Biosystems) and *RNU48* (assay ID: 001006; Applied Biosystems), respectively. The fold-change was calculated using the delta–delta Ct method.

### Transfection with mature miRNA and small interfering RNA (si-RNA) by transfection of lung SCC cells

Here, we used the following miRNA species: Pre-miR™ miRNA precursors (hsa-*miR-145-5p*, assay ID: PM 11480; hsa-*miR-145-3p*, assay ID: PM 13036; negative control miRNA; assay ID: AM 17111) (Thermo Fisher). We also purchased the following from Invitrogen (Carlsbad, CA, USA): Stealth Select RNAi siRNA, si-*MTDH* (assay ID: HSS 150644, and P/N: HSS 150646. Thermo Fisher Scientific (Waltham, MA USA) provided negative-control siRNA (D-001810-10). For transfection, RNAs were incubated with OPTI-MEM (Invitrogen) and Lipofectamine RNAiMax reagent (Invitrogen) as previously studies [12–17].

### Cell proliferation, migration, and invasion assays

XTT assays were used to assess cell proliferation (Cell Proliferation Kit II, Roche Applied Sciences, Tokyo, Japan). Cell migration was analysed with wound healing assays. Modified Boyden chambers containing Transwell-precoated Matrigel membrane filter inserts were used to quantitate cellular invasion. Details of these assays were described as previously [12–17].

### Use of total genome expression and *in silico* analyses to identify genes regulated by *miR-145-5p* and *miR-145-3p* in lung SCC cells

Specific genes affected by *miR-145-5p* and *miR-145-3p* were identified by a combination of *in silico* and genome-wide gene expression analyses. Genes regulated by *miR-145-5p* and *miR-145-3p* were listed using the TargetScan database. We then attempted to identify targets using the EBC-1 cell line transfected with these miRNAs. A Sure Print G3 Human GE 8 × 60K Microarray (Agilent Technologies) was used for expression profiling of *miR-145-5p* and *miR-145-3p* transfectants. The microarray data were deposited into GEO (http://www.ncbi.nlm.nih.gov/geo/), accession number GSE77790. Genes upregulated in NSCLC were obtained from publicly available data sets in GEO (accession numbers: GSE19188). The overall strategy is outlined in Figure 2.

### Regulation of targets downstream from *MTDH* in lung SCC

We investigated lung SCC cells to identify pathways regulated by *MTDH*. We analyzed gene expression using *si-MTDH*-transfected EBC-1 cells. Microarray data were used for expression profiling of si-*MTDH* transfectants. The microarray data were deposited into GEO (accession number: GSE77790). We analyzed common down- or upregulated genes using the GEO dataset.

### Western blotting

Protein lysates (50 μg) were obtained 96 h after transfection. Proteins were separated by NuPAGE on 4–12% bis-tris gels (Invitrogen) and transferred to polyvinylidene fluoride membranes. Membranes were immunoblotted with diluted polyclonal MDTH antibody (1:200; HPA 015104; Sigma-Aldrich, St Lois, MO, USA) and GAPDH antibody (1:100; MAB374; Chemicon, Temecula, CA, USA). The procedure of Western blotting was described previously [12–17].

### Plasmid construction and dual-luciferase reporter assay

The partial wild-type sequence of the *MTDH* 3′-untranslated region (UTR) was inserted between the XhoI–PmeI restriction sites in the 3′-UTR of the *hRluc* gene in the psiCHECK-2 vector (C8021; Promega, Madison, WI, USA). Alternatively, we used sequences that were missing the *miR-145-5p* target sites (position 3151-3157 or position 3503-3510) or *the miR-145-3p* target site (position 147-153). The synthesized DNA was cloned into the psiCHECK-2 vector. EBC-1 cells were transfected with 20 ng of the vector, 20 nM microRNAs, and 1 μL Lipofectamine 2000 (Invitrogen) in 100 μL Opti-MEM (Invitrogen). The procedure of dual-luciferase reporter assay was described previously [12–17].

### TCGA database analysis of lung SCC specimens

The clinical significance of *MTDH* in lung SCC was assessed by RNA sequencing and a putative CNV database (predicted by a GISTIC algorithm) in LUSCC TCGA (The Cancer Genome Atlas: https://tcga-data.nci.nih.gov/). The genomic and clinical data were retrieved from cBioportal (http://www.cbioportal.org/) or UCSC Cancer Browser (https://genome-cancer.ucsc.edu/proj/site/hgHeatmap/). The normalized mRNA expression values in the RNA sequencing data were processed and distributed in log_2_ transformed RSEM (RNA-Seq by Expectation Maximization) values (cBioportal) or log_2_ transformed (RSEM+1) (UCSC Cancer Browser). The Z-scores of *MTDH* mRNA expression data and clinical sample information corresponding to LUSCC patients were collected from cBioportal. The *MTDH* altered group (CNV = amplification or gain, or mRNA Z-score > 1.5, total 313 cases) and the *MTDH* non-altered group (CNV = diploid or heterozygous loss, and mRNA Z-score ≤ 1.5, total 184 cases) were analyzed by Kaplan–Meier survival curves and log-rank statistics.

### Statistical analysis

The relationships between 2 groups and the numerical values obtained by RT-qPCR were analysed using Mann-Whitney U-tests. Spearman's rank test was used to evaluate the correlations between the expression of *miR-145-5p* and *miR-145-3p*. The relationships among more than 3 variables and numerical values were analysed using the Bonferroni-adjusted Mann-Whitney U-test. Survival analysis was analysed by the Kaplan-Meier method and log-rank test, using Stat Mate software (version 4.01, ATMS Co., Tokyo, Japan). All other analyses were performed using Expert StatView (version 5, SAS Institute Inc., Cary, NC, USA).

## SUPPLEMENTARY FIGURES AND TABLES






