# Supplementary material for: Dual-strand tumor-suppressor microRNA-145 (miR-145-5p and miR-145-3p) coordinately targeted MTDH in lung squamous cell carcinoma
Source: Oncotarget. 2016 Sep 27;7(44):72084–98. doi: 10.18632/oncotarget.12290 (PMC5342147; doi:10.18632/oncotarget.12290)
Supplement: Supplementary file 1 [file oncotarget-07-72084-s001.pdf]

Dual-strand tumor-suppressor *microRNA-145* (*miR-145-5p* and *miR-145-3p*) coordinately targeted *MTDH* in lung squamous cell carcinoma

SUPPLEMENTARY FIGURES AND TABLES

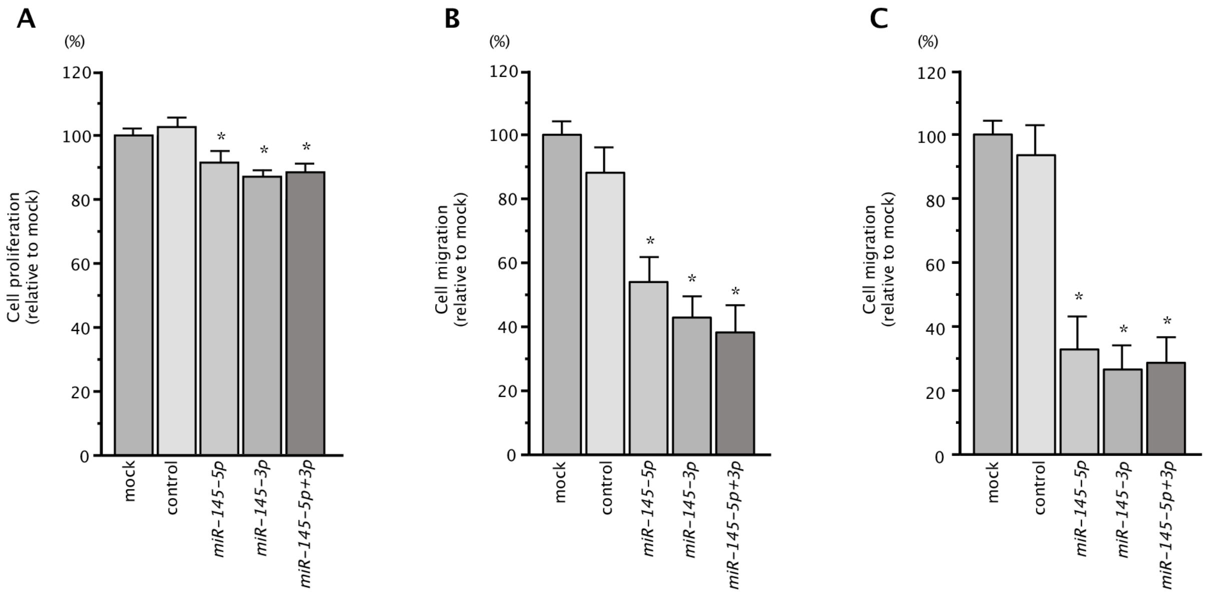

Supplementary Figure S1: Effects of co-transfection of *miR-145-5p* and *miR-145-3p* on cell proliferation, migration and invasion assays. Co-transfection of *miR-145-5p* (10 nM) and *miR-145-3p* (10 nM) did not show synergistic effects on cancer cell aggressiveness compared with *miR-145-5p* or *miR-145-3p* transfection independently in EBC-1 cells.

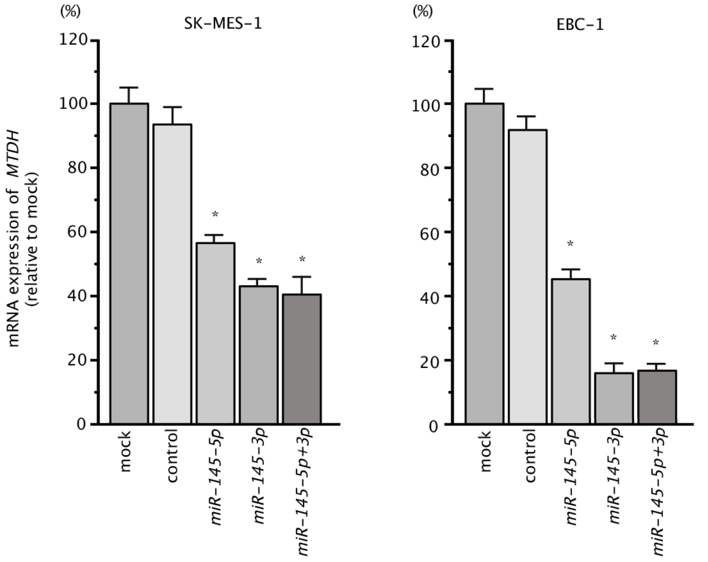

Supplementary Figure S2: Effects of co-transfection of *miR-145-5p* (10 nM) and *miR-145-3p* (10 nM) on expression levels of *MTDH* in SK-MES-1 and EBC-1 cells.

Supplementary Table S1A: Downregulated genes by si-*MTDH* (si-*MTDH*-1) in EBC-1 cells.

Supplementary Table S1B: Downregulated genes by si-*MTDH* (si-*MTDH*-2) in EBC-1 cells.

See Supplementary File 1

Supplementary Table S2: Downregulated genes in si-*MTDH* transfectant.

See Supplementary File 2
