# Supplementary material for: Dual-strand tumor-suppressor microRNA-145 (miR-145-5p and miR-145-3p) coordinately targeted MTDH in lung squamous cell carcinoma
Source: Oncotarget. 2016 Sep 27;7(44):72084–98. doi: 10.18632/oncotarget.12290 (PMC5342147; doi:10.18632/oncotarget.12290)
Supplement: Supplementary file 2 [file oncotarget-07-72084-s002.docx]

| Supplementary Table1A: Downregulated genes by si-*MTDH (*si-*MTDH*-1*)* in EBC-1 cells | | |  |
| --- | --- | --- | --- |
| Entrez gene ID | Gene symbol | Gene name | si-*MTDH* transfectant (fold-change) |
|  |  |  |  |
| 7056 | *THBD* | thrombomodulin | -19.37 |
| 5104 | *SERPINA5* | serpin peptidase inhibitor, clade A (alpha-1 antiproteinase, antitrypsin), member 5 | -15.49 |
| 8839 | *WISP2* | WNT1 inducible signaling pathway protein 2 | -15.45 |
| 983 | *CDK1* | cyclin-dependent kinase 1 | -15.41 |
| 1673 | *DEFB4A* | defensin, beta 4A | -13.57 |
| 11031 | *RAB31* | RAB31, member RAS oncogene family | -12.45 |
| 5230 | *PGK1* | phosphoglycerate kinase 1 | -11.56 |
| 26227 | *PHGDH* | phosphoglycerate dehydrogenase | -11.32 |
| 140809 | *SRXN1* | sulfiredoxin 1 | -10.43 |
| 334 | *APLP2* | amyloid beta (A4) precursor-like protein 2 | -10.19 |
| 5328 | *PLAU* | plasminogen activator, urokinase | -9.77 |
| 84176 | *MYH16* | myosin, heavy chain 16 pseudogene | -9.72 |
| 6185 | *RPN2* | ribophorin II | -9.51 |
| 57761 | *TRIB3* | tribbles pseudokinase 3 | -9.49 |
| 629 | *CFB* | complement factor B | -9.16 |
| 11113 | *CIT* | citron rho-interacting serine/threonine kinase | -9.14 |
| 344887 | *LOC344887* | NmrA-like family domain containing 1 pseudogene | -9.11 |
| 6289 | *SAA2* | serum amyloid A2 | -8.95 |
| 8614 | *STC2* | stanniocalcin 2 | -8.90 |
| 3122 | *HLA-DRA* | major histocompatibility complex, class II, DR alpha | -8.48 |
| 950 | *SCARB2* | scavenger receptor class B, member 2 | -8.46 |
| 3303 | *HSPA1A* | heat shock 70kDa protein 1A | -8.28 |
| 3127 | *HLA-DRB5* | major histocompatibility complex, class II, DR beta 5 | -8.22 |
| 3553 | *IL1B* | interleukin 1, beta | -7.96 |
| 51292 | *GMPR2* | guanosine monophosphate reductase 2 | -7.88 |
| 3109 | *HLA-DMB* | major histocompatibility complex, class II, DM beta | -7.84 |
| 4778 | *NFE2* | nuclear factor, erythroid 2 | -7.76 |
| 8030 | *CCDC6* | coiled-coil domain containing 6 | -7.46 |
| 388524 | *RPSAP58* | ribosomal protein SA pseudogene 58 | -7.45 |
| 92140 | *MTDH* | metadherin | -7.36 |
| 4490 | *MT1B* | metallothionein 1B | -7.24 |
| 388524 | *RPSAP58* | ribosomal protein SA pseudogene 58 | -7.22 |
| 1827 | *RCAN1* | regulator of calcineurin 1 | -7.18 |
| 1305 | *COL13A1* | collagen, type XIII, alpha 1 | -7.17 |
| 6185 | *RPN2* | ribophorin II | -7.16 |
| 100505994 | *LUCAT1* | lung cancer associated transcript 1 (non-protein coding) | -7.09 |
| 404636 | *FAM45A* | family with sequence similarity 45, member A | -7.06 |
| 388962 | *BOLA3* | bolA family member 3 | -7.05 |
| 29968 | *PSAT1* | phosphoserine aminotransferase 1 | -7.03 |
| 6891 | *TAP2* | transporter 2, ATP-binding cassette, sub-family B (MDR/TAP) | -6.83 |
|  |  |  |  |
| Supplementary Table 1B: Downregulated genes by si-*MTDH (*si-*MTDH*-2*)* in EBC-1 cells | | |  |
| Entrez gene ID | Gene symbol | Gene name | si-*MTDH* transfectant (fold-change) |
|  |  |  |  |
| 6273 | *S100A2* | S100 calcium binding protein A2 | -7.53 |
| 1645 | *AKR1C1* | aldo-keto reductase family 1, member C1 | -7.49 |
| 79803 | *HPS6* | Hermansky-Pudlak syndrome 6 | -7.07 |
| 10156 | *RASA4* | RAS p21 protein activator 4 | -6.34 |
| 340335 | *LOC340335* | uncharacterized LOC340335 | -6.13 |
| 1645 | *AKR1C1* | aldo-keto reductase family 1, member C1 | -5.90 |
| 6279 | *S100A8* | S100 calcium binding protein A8 | -5.78 |
| 92140 | *MTDH* | metadherin | -5.57 |
| 375775 | *PNPLA7* | patatin-like phospholipase domain containing 7 | -5.57 |
| 7627 | *ZNF75A* | zinc finger protein 75a | -5.54 |
| 26048 | *ZNF500* | zinc finger protein 500 | -5.48 |
| 6604 | *SMARCD3* | SWI/SNF related, matrix associated, actin dependent regulator of chromatin, subfamily d, member 3 | -5.31 |
| 26007 | *DAK* | dihydroxyacetone kinase 2 homolog (S. cerevisiae) | -5.28 |
| 116412 | *ZNF837* | zinc finger protein 837 | -5.16 |
| 100289187 | *GS1-259H13.2* | transmembrane protein 225-like | -5.14 |
| 79789 | *CLMN* | calmin (calponin-like, transmembrane) | -5.13 |
| 100505994 | *LUCAT1* | lung cancer associated transcript 1 (non-protein coding) | -5.08 |
| 100507297 | *TMEM44-AS1* | TMEM44 antisense RNA 1 | -5.07 |
| 135228 | *CD109* | CD109 molecule | -4.96 |
| 3251 | *HPRT1* | hypoxanthine phosphoribosyltransferase 1 | -4.90 |
| 4778 | *NFE2* | nuclear factor, erythroid 2 | -4.72 |
| 100289187 | *GS1-259H13.2* | transmembrane protein 225-like | -4.71 |
| 4287 | *ATXN3* | ataxin 3 | -4.70 |
| 100653022 | *PP12719* | uncharacterized LOC100653022 | -4.69 |
| 100505994 | *LUCAT1* | lung cancer associated transcript 1 (non-protein coding) | -4.69 |
| 56649 | *TMPRSS4* | transmembrane protease, serine 4 | -4.66 |
| 93273 | *LEMD1* | LEM domain containing 1 | -4.63 |
| 7349 | *UCN* | urocortin | -4.55 |
| 90853 | *SPOCD1* | SPOC domain containing 1 | -4.52 |
| 117155 | *CATSPER2* | cation channel, sperm associated 2 | -4.49 |
| 57654 | *UVSSA* | UV-stimulated scaffold protein A | -4.37 |
| 116844 | *LRG1* | leucine-rich alpha-2-glycoprotein 1 | -4.36 |
| 6590 | *SLPI* | secretory leukocyte peptidase inhibitor | -4.35 |
| 266977 | *GPR110* | G protein-coupled receptor 110 | -4.30 |
| 3553 | *IL1B* | interleukin 1, beta | -4.28 |
| 8829 | *NRP1* | neuropilin 1 | -4.20 |
| 23097 | *CDK19* | cyclin-dependent kinase 19 | -4.17 |
| 4157 | *MC1R* | melanocortin 1 receptor (alpha melanocyte stimulating hormone receptor) | -4.16 |
| 8714 | *ABCC3* | ATP-binding cassette, sub-family C (CFTR/MRP), member 3 | -4.12 |
| 1954 | *MEGF8* | multiple EGF-like-domains 8 | -4.07 |
