# Supplementary material for: Dual-strand tumor-suppressor microRNA-145 (miR-145-5p and miR-145-3p) coordinately targeted MTDH in lung squamous cell carcinoma
Source: Oncotarget. 2016 Sep 27;7(44):72084–98. doi: 10.18632/oncotarget.12290 (PMC5342147; doi:10.18632/oncotarget.12290)
Supplement: Supplementary file 3 [file oncotarget-07-72084-s003.docx]

| **Supplemental Table 2: Downregulated genes in si-*MTDH* transfectant** | | | | |
| --- | --- | --- | --- | --- |
| KEGG entry no. | Annotations | No. of genes | P-value | Genes |
| Kegg:04510 | Focal adhesion | 31 | 2.20E-07 | *CAV2,MET,ITGA6,TNR,ERBB2,ROCK2,PTEN,FLNA,PXN,ITGB6,AKT3,PRKCA,TNXB,LAMC1,CAV1,ARHGAP5,LAMB3,EGFR,PIK3R1,CAV3,TNC,LAMC2,ITGB1,COL5A1,CTNNB1,VEGFA,AKT2,THBS3,PPP1CC,PPP1CB,FLNB* |
| Kegg:05145 | Toxoplasmosis | 22 | 2.67E-06 | *HLA-DMA,IFNGR2,IKBKB,CYCS,ITGA6,HLA-DRB5,TNFRSF1A,AKT3,ALOX5,MYD88,LAMC1,LY96,LAMB3,HLA-DPB1,IFNGR1,PIK3R1,LAMC2,ITGB1,HLA-DRB1,HLA-DQB1,HLA-DMB,AKT2* |
| Kegg:04114 | Oocyte meiosis | 20 | 5.72E-06 | *PTTG2,ESPL1,CCNB2,ADCY6,PTTG1,YWHAQ,CCNB1,REC8,ADCY3,PPP2R5C,RPS6KA2,PPP2R1A,MAD2L2,ANAPC11,BUB1,RPS6KA3,AURKA,CDK1,PPP1CC,PPP1CB* |
| Kegg:04612 | Antigen processing and presentation | 14 | 6.14E-06 | *HLA-DMA,CANX,HSPA5,HLA-DRB5,CD74,CTSB,HLA-DPB1,PDIA3,KLRC4,LGMN,HLA-DRB1,HLA-DQB1,HLA-DMB,RFX5* |
| Kegg:04141 | Protein processing in endoplasmic reticulum | 23 | 2.37E-05 | *NSFL1C,UBE2J1,SEC24C,CANX,HYOU1,HSPA5,GANAB,RPN2,DDIT3,MAN1B1,ATXN3,SEC31B,ERO1L,PDIA3,UBE2D4,PDIA4,DNAJC3,SSR3,HSP90B1,EIF2S1,RAD23B,DNAJB12,SSR1* |
| Kegg:00230 | Purine metabolism | 23 | 2.54E-05 | *POLD3,ADCY6,HPRT1,GMPR2,PDE6D,ENPP1,NME4,NUDT5,POLR3H,NME7,RRM1,ADCY3,ADA,POLD1,ADSL,NME3,POLR2K,ENTPD2,PRIM1,GMPR,NT5E,NT5M,XDH* |
| Kegg:05200 | Pathways in cancer | 35 | 4.40E-05 | *IKBKB,MMP9,CYCS,NCOA4,EGLN3,MET,ITGA6,EPAS1,ERBB2,CCDC6,PTEN,RAD51,BCR,AKT3,RUNX1,PRKCA,LAMC1,LAMB3,EGFR,FZD2,PIK3R1,MYC,CKS1B,LAMC2,ITGB1,SMAD4,APPL1,HSP90B1,SLC2A1,CTNNB1,PLD1,VEGFA,RXRA,PPARG,AKT2* |
| Kegg:04144 | Endocytosis | 25 | 4.82E-05 | *CAV2,MET,VPS36,ASAP3,RAB5B,SH3GLB1,RAB11A,IQSEC1,ARAP3,ITCH,EHD2,NEDD4L,RAB11FIP1,GIT2,GRK5,CAV1,PIP5KL1,EGFR,CAV3,RAB31,AP2B1,PLD1,EPN2,SMURF1,EPN3* |
| Kegg:05152 | Tuberculosis | 23 | 6.32E-05 | *IL1B,HLA-DMA,IFNGR2,CYCS,C3,HLA-DRB5,RAB5B,CD74,TNFRSF1A,IRAK2,AKT3,MYD88,HLA-DPB1,IFNGR1,CEBPG,TRADD,MRC2,HLA-DRB1,HLA-DQB1,HLA-DMB,LSP1,AKT2,RFX5* |
| Kegg:04110 | Cell cycle | 18 | 0.000191356 | *PTTG2,ESPL1,CCNB2,CCNA2,PTTG1,CDC7,YWHAQ,CHEK2,CCNB1,ORC4,WEE1,MYC,MAD2L2,BUB1B,ANAPC11,SMAD4,BUB1,CDK1* |
| Kegg:04940 | Type I diabetes mellitus | 9 | 0.000250847 | *IL1B,HLA-DMA,ICA1,HLA-DRB5,CD86,HLA-DPB1,HLA-DRB1,HLA-DQB1,HLA-DMB* |
| Kegg:00240 | Pyrimidine metabolism | 15 | 0.000267505 | *TYMS,POLD3,NME4,AK3,POLR3H,NME7,RRM1,POLD1,DPYD,NME3,POLR2K,PRIM1,NT5E,TK1,NT5M* |
| Kegg:05222 | Small cell lung cancer | 14 | 0.000321544 | *IKBKB,CYCS,ITGA6,PTEN,AKT3,LAMC1,LAMB3,PIK3R1,MYC,CKS1B,LAMC2,ITGB1,RXRA,AKT2* |
| Kegg:04914 | Progesterone-mediated oocyte maturation | 14 | 0.000366749 | *CCNB2,ADCY6,CCNA2,CCNB1,AKT3,ADCY3,RPS6KA2,PIK3R1,MAD2L2,ANAPC11,BUB1,RPS6KA3,AKT2,CDK1* |
| Kegg:04210 | Apoptosis | 14 | 0.000366749 | *IL1B,IKBKB,CYCS,TNFRSF10B,ENDOD1,TNFRSF1A,IRAK2,AKT3,MYD88,IL1RAP,PIK3R1,TRADD,AKT2,RIPK1* |
| Kegg:05140 | Leishmaniasis | 12 | 0.000413929 | *IL1B,HLA-DMA,IFNGR2,C3,HLA-DRB5,MYD88,HLA-DPB1,IFNGR1,ITGB1,HLA-DRB1,HLA-DQB1,HLA-DMB* |
| Kegg:05332 | Graft-versus-host disease | 8 | 0.000440295 | *IL1B,HLA-DMA,HLA-DRB5,CD86,HLA-DPB1,HLA-DRB1,HLA-DQB1,HLA-DMB* |
| Kegg:04115 | p53 signaling pathway | 12 | 0.000455476 | *CYCS,CCNB2,TNFRSF10B,IGFBP3,PTEN,CHEK2,CCNB1,SESN2,SERPINE1,STEAP3,MDM4,CDK1* |
| Kegg:00750 | Vitamin B6 metabolism | 4 | 0.000615479 | *PNPO,PHOSPHO2,PSAT1,PDXK* |
| Kegg:04150 | mTOR signaling pathway | 10 | 0.000831844 | *RPTOR,AKT3,EIF4E2,RPS6KA2,PIK3R1,DDIT4,ULK1,RPS6KA3,VEGFA,AKT2* |
| Kegg:04142 | Lysosome | 16 | 0.000863024 | *NAGPA,SCARB2,CTSF,CTSC,CTSA,GALC,LIPA,GAA,AP3M2,LAMP3,CTSB,GNS,LGMN,LAPTM4B,HGSNAT,GGA2* |
| Kegg:04512 | ECM-receptor interaction | 13 | 0.000866186 | *ITGA6,TNR,ITGB6,TNXB,LAMC1,LAMB3,TNC,LAMC2,ITGB1,CD44,CD47,COL5A1,THBS3* |
| Kegg:00010 | Glycolysis / Gluconeogenesis | 11 | 0.000887898 | *PGK1,ADH5,ALDH1A3,ENO3,LDHA,ALDH9A1,ENO2,GAPDH,ADPGK,TPI1,PCK2* |
| Kegg:05416 | Viral myocarditis | 11 | 0.00089806 | *HLA-DMA,CYCS,HLA-DRB5,SGCB,CD86,CAV1,HLA-DPB1,ICAM1,HLA-DRB1,HLA-DQB1,HLA-DMB* |
| Kegg:04120 | Ubiquitin mediated proteolysis | 17 | 0.00090508 | *UBE2C,UBE2J1,PRPF19,DET1,HERC4,ITCH,UBE2R2,FBXW8,BRCA1,NEDD4L,SOCS3,KEAP1,UBE2Q2,ANAPC11,RHOBTB1,UBE2D4,SMURF1* |
| Kegg:03440 | Homologous recombination | 7 | 0.000915966 | *POLD3,RAD51,EME1,POLD1,RAD51C,RAD54L,BLM* |
| Kegg:05130 | Pathogenic Escherichia coli infection | 10 | 0.000945623 | *YWHAQ,WAS,ROCK2,PRKCA,LY96,ARPC1A,TUBA1B,ITGB1,ARHGEF2,CTNNB1* |
| Kegg:04145 | Phagosome | 17 | 0.000973877 | *HLA-DMA,C3,CANX,ATP6V1C1,HLA-DRB5,RAB5B,ATP6V1A,HLA-DPB1,TUBA1B,ITGB1,MRC2,HLA-DRB1,VAMP3,HLA-DQB1,SCARB1,HLA-DMB,THBS3* |
| Kegg:04070 | Phosphatidylinositol signaling system | 12 | 0.000988943 | *CDS1,DGKA,CDS2,OCRL,INPPL1,PIK3C2B,PTEN,PRKCA,ITPKA,INPP5E,PIK3R1,CDIPT* |
| Kegg:04920 | Adipocytokine signaling pathway | 11 | 0.00118152 | *IKBKB,PTPN11,LEPR,TNFRSF1A,AKT3,SOCS3,TRADD,SLC2A1,RXRA,AKT2,PCK2* |
| Kegg:05150 | Staphylococcus aureus infection | 9 | 0.00119313 | *HLA-DMA,C3,C1S,HLA-DRB5,HLA-DPB1,ICAM1,HLA-DRB1,HLA-DQB1,HLA-DMB* |
| Kegg:05330 | Allograft rejection | 7 | 0.00151009 | *HLA-DMA,HLA-DRB5,CD86,HLA-DPB1,HLA-DRB1,HLA-DQB1,HLA-DMB* |
| Kegg:05100 | Bacterial invasion of epithelial cells | 11 | 0.00165433 | *CAV2,MET,WAS,PXN,CAV1,ARPC1A,PIK3R1,CAV3,MAD2L2,ITGB1,CTNNB1* |
| Kegg:05160 | Hepatitis C | 16 | 0.00185042 | *IKBKB,TNFRSF1A,AKT3,SOCS3,MAVS,EGFR,PIK3R1,PPP2R1A,TRADD,SCARB1,EIF2S1,RXRA,AKT2,RIPK1,IRF9,NR1H3* |
| Kegg:05323 | Rheumatoid arthritis | 12 | 0.00207752 | *IL1B,HLA-DMA,ATP6V1C1,HLA-DRB5,ATP6V1A,CD86,HLA-DPB1,ICAM1,HLA-DRB1,HLA-DQB1,VEGFA,HLA-DMB* |
| Kegg:04146 | Peroxisome | 11 | 0.00314634 | *AGPS,PEX19,ACOX1,PRDX1,PEX7,PEX6,PEX1,MPV17,DHRS4,PEX13,XDH* |
| Kegg:05310 | Asthma | 6 | 0.00371345 | *HLA-DMA,HLA-DRB5,HLA-DPB1,HLA-DRB1,HLA-DQB1,HLA-DMB* |
| Kegg:04962 | Vasopressin-regulated water reabsorption | 8 | 0.00372587 | *DCTN1,AQP3,ADCY6,CREB3L2,RAB5B,RAB11A,ADCY3,STX4* |
| Kegg:03030 | DNA replication | 7 | 0.00422595 | *RNASEH2A,POLD3,LIG1,RFC2,POLD1,PRIM1,RFC4* |
| Kegg:00562 | Inositol phosphate metabolism | 9 | 0.0047101 | *OCRL,INPPL1,PIK3C2B,PTEN,ITPKA,PIP5KL1,INPP5E,CDIPT,TPI1* |
| Kegg:00051 | Fructose and mannose metabolism | 7 | 0.00479999 | *AKR1B10,FUK,AKR1B1,PMM2,MTMR1,TPI1,PFKFB4* |
| Kegg:03320 | PPAR signaling pathway | 10 | 0.00516686 | *SLC27A1,ACOX1,CPT2,ANGPTL4,RXRA,PPARG,SCD5,ME1,PCK2,NR1H3* |
| Kegg:05212 | Pancreatic cancer | 10 | 0.00516686 | *IKBKB,ERBB2,RAD51,AKT3,EGFR,PIK3R1,SMAD4,PLD1,VEGFA,AKT2* |
| Kegg:05216 | Thyroid cancer | 6 | 0.00608486 | *NCOA4,CCDC6,MYC,CTNNB1,RXRA,PPARG* |
| Kegg:04630 | Jak-STAT signaling pathway | 16 | 0.00637722 | *IFNGR2,PTPN11,LEPR,AKT3,SOCS3,IL6ST,JAK3,IFNGR1,PIK3R1,MYC,IL7,IL4R,IL11RA,AKT2,IRF9,IL13RA1* |
| Kegg:00510 | N-Glycan biosynthesis | 8 | 0.00640265 | *MGAT5,GANAB,MAN2A2,RPN2,MAN1B1,MGAT2,ST6GAL1,B4GALT2* |
| Kegg:00620 | Pyruvate metabolism | 7 | 0.00672472 | *LDHD,LDHA,ALDH9A1,AKR1B1,ME1,PCK2,PC* |
| Kegg:04380 | Osteoclast differentiation | 14 | 0.00682188 | *IL1B,IFNGR2,IKBKB,SIRPA,TNFRSF1A,FHL2,AKT3,SOCS3,IFNGR1,PIK3R1,NFATC1,PPARG,AKT2,IRF9* |
| Kegg:00020 | Citrate cycle (TCA cycle) | 6 | 0.00766784 | *IDH3B,SDHC,IDH3A,DLST,PCK2,PC* |
| Kegg:00970 | Aminoacyl-tRNA biosynthesis | 7 | 0.00768157 | *IARS,WARS,SARS,AARS,CARS2,AARS2,CARS* |
| Kegg:05322 | Systemic lupus erythematosus | 11 | 0.00779381 | *HLA-DMA,C3,C1S,H2AFX,HLA-DRB5,CD86,H2AFY,HLA-DPB1,HLA-DRB1,HLA-DQB1,HLA-DMB* |
| Kegg:05162 | Measles | 14 | 0.00799485 | *IL1B,IFNGR2,TNFRSF10B,AKT3,MYD88,CSNK2A2,JAK3,MAVS,DOK1,IFNGR1,PIK3R1,EIF2S1,AKT2,IRF9* |
| Kegg:05142 | Chagas disease (American trypanosomiasis) | 12 | 0.00803588 | *IL1B,IFNGR2,IKBKB,C3,TNFRSF1A,AKT3,MYD88,SERPINE1,IFNGR1,PIK3R1,PPP2R1A,AKT2* |
| Kegg:05213 | Endometrial cancer | 8 | 0.00806352 | *ERBB2,PTEN,AKT3,EGFR,PIK3R1,MYC,CTNNB1,AKT2* |
| Kegg:00532 | Glycosaminoglycan biosynthesis - chondroitin sulfate | 5 | 0.00945031 | *CHST12,CHST15,CSGALNACT1,B3GALT6,DSE* |
| Kegg:04910 | Insulin signaling pathway | 14 | 0.00954458 | *EXOC7,IKBKB,GYS1,RPTOR,AKT3,SOCS3,SREBF1,EIF4E2,PIK3R1,AKT2,PCK2,PYGL,PPP1CC,PPP1CB* |
| Kegg:00564 | Glycerophospholipid metabolism | 10 | 0.00987264 | *CDS1,DGKA,CDS2,LPCAT3,GPD1L,PLB1,AGPAT2,CDIPT,PLD1,PNPLA7* |
| Kegg:03430 | Mismatch repair | 5 | 0.011035 | *POLD3,LIG1,RFC2,POLD1,RFC4* |
| Kegg:04672 | Intestinal immune network for IgA production | 7 | 0.011235 | *HLA-DMA,HLA-DRB5,CD86,HLA-DPB1,HLA-DRB1,HLA-DQB1,HLA-DMB* |
| Kegg:05320 | Autoimmune thyroid disease | 7 | 0.012588 | *HLA-DMA,HLA-DRB5,CD86,HLA-DPB1,HLA-DRB1,HLA-DQB1,HLA-DMB* |
| Kegg:05211 | Renal cell carcinoma | 9 | 0.0133337 | *EGLN3,MET,PTPN11,EPAS1,AKT3,PIK3R1,SLC2A1,VEGFA,AKT2* |
| Kegg:04520 | Adherens junction | 9 | 0.0144413 | *MET,ERBB2,WAS,CSNK2A2,EGFR,SMAD4,SNAI1,BAIAP2,CTNNB1* |
| Kegg:05220 | Chronic myeloid leukemia | 9 | 0.017121 | *IKBKB,PTPN11,BCR,AKT3,RUNX1,PIK3R1,MYC,SMAD4,AKT2* |
| Kegg:05215 | Prostate cancer | 10 | 0.0192461 | *IKBKB,CREB3L2,ERBB2,PTEN,AKT3,EGFR,PIK3R1,HSP90B1,CTNNB1,AKT2* |
| Kegg:05210 | Colorectal cancer | 8 | 0.0199852 | *CYCS,AKT3,PIK3R1,MYC,SMAD4,APPL1,CTNNB1,AKT2* |
| Kegg:00670 | One carbon pool by folate | 4 | 0.0231738 | *TYMS,SHMT2,MTHFD2,AMT* |
| Kegg:04978 | Mineral absorption | 7 | 0.0231815 | *MT2A,MT1F,MT1M,MT1E,MT1X,HMOX1,MT1B* |
| Kegg:04666 | Fc gamma R-mediated phagocytosis | 10 | 0.0246881 | *ASAP3,WAS,CFL2,AKT3,PRKCA,LYN,ARPC1A,PIK3R1,PLD1,AKT2* |
| Kegg:04610 | Complement and coagulation cascades | 8 | 0.0250097 | *THBD,C3,C1S,SERPINA1,SERPINE1,SERPINA5,TFPI,PLAU* |
| Kegg:05223 | Non-small cell lung cancer | 7 | 0.0269539 | *ERBB2,AKT3,PRKCA,EGFR,PIK3R1,RXRA,AKT2* |
| Kegg:03420 | Nucleotide excision repair | 6 | 0.0275232 | *POLD3,LIG1,RFC2,POLD1,RAD23B,RFC4* |
| Kegg:04514 | Cell adhesion molecules (CAMs) | 12 | 0.0299102 | *HLA-DMA,ITGA6,HLA-DRB5,NLGN2,CD86,HLA-DPB1,ICAM1,ITGB1,HLA-DRB1,PVR,HLA-DQB1,HLA-DMB* |
| Kegg:00740 | Riboflavin metabolism | 3 | 0.0325234 | *ENPP1,ACP6,BLVRB* |
| Kegg:00260 | Glycine, serine and threonine metabolism | 5 | 0.036689 | *SHMT2,PHGDH,AMT,PSAT1,CBS* |
| Kegg:01040 | Biosynthesis of unsaturated fatty acids | 4 | 0.0369098 | *ACOX1,TECR,SCD5,ELOVL6* |
| Kegg:03410 | Base excision repair | 5 | 0.0406078 | *POLD3,LIG1,NEIL1,POLD1,MBD4* |
| Kegg:05010 | Alzheimer's disease | 14 | 0.040916 | *IL1B,CYCS,MAPT,TNFRSF1A,NDUFA12,GRIN2D,BACE1,SDHC,NDUFV3,BACE2,APP,APOE,GAPDH,UQCRQ* |
| Kegg:05146 | Amoebiasis | 10 | 0.0428155 | *IL1B,ARG2,RAB5B,SERPINB1,PRKCA,LAMC1,LAMB3,PIK3R1,LAMC2,COL5A1* |
| Kegg:04310 | Wnt signaling pathway | 13 | 0.0435157 | *SENP2,ROCK2,PRKCA,CSNK2A2,PPP2R5C,FZD2,PPP2R1A,MYC,CTNNBIP1,TBL1XR1,SMAD4,CTNNB1,NFATC1* |
| Kegg:04320 | Dorso-ventral axis formation | 4 | 0.0470815 | *SPIRE2,NOTCH3,EGFR,SPIRE1* |
| Kegg:05131 | Shigellosis | 7 | 0.0485925 | *IKBKB,WAS,ROCK2,ARPC1A,MAD2L2,ITGB1,CD44* |
